# Isolation and structure elucidation of constituents of *Citrus limon*, *Isodon japonicus*, and *Lansium domesticum* as the cancer prevention agents

**DOI:** 10.1186/s41021-020-00156-0

**Published:** 2020-04-16

**Authors:** Takahiro Matsumoto, Tetsushi Watanabe

**Affiliations:** Department of Public Health, Kyoto Pharmaceutical University, Misasagi, Yamashina-ku, Kyoto, 607-8412 Japan

**Keywords:** *Citrus limon*, *Isodon japonicus*, *Lansium domesticum*, Antimutagenic effects, Ames test, Micronucleus test

## Abstract

In the course of our research to investigate the cancer prevention potency of natural products derived from plant materials, we isolated fifty-five compounds, including twenty-one new compounds from the peels of *Citrus limon*, aerial parts of *Isodon japonicus*, and leaves of *Lansium domesticum.* The chemical structures of the isolated compounds were elucidated by chemical/physicochemical evidence, and nuclear magnetic resonance spectroscopy and mass spectrometry results. Moreover, the absolute stereochemistry of the new compounds were elucidated by various techniques such as chemical synthesis, modified Mosher’s method, Cu-Kα X-ray crystallographic analysis, and comparison of experimental and predicted electronic circular dichroism data. The antimutagenic effects of the isolated and structure-elucidated compounds against heterocyclic amines, 3-amino-1,4-dimethyl-5*H*-pyrido [4,3-*b*]indole (Trp-P-1) and 2-amino-1-methyl-6-phenylimidazo[4,5-*b*]pyridine (PhIP), were evaluated by the Ames test and in vivo micronucleus test. In this review, we present the comprehensive results of the antimutagenic effects of the isolated natural products.

## Background

Cancer is the leading cause of death worldwide and one of the major risk factors is exposure to the agents that damage the genetic material. These agents are known as genotoxins and, according to their mode of action, are classified into mutagens, carcinogens, or teratogens [1, 2]. Trp-P-1 and PhIP are well known mutagenic and carcinogenic heterocyclic amines that are found in cooked meat. Therefore, it is difficult to completely avoid these risk factors in daily life. On the other hand, previous case-control studies have suggested that the consumption of some plant derived foods such as citrus fruits is associated with a reduced all-cancer incidence [3].

Based on these studies, we searched for antimutagenic materials derived from foods. In the course of this study, we found using the Ames test that the methanolic (MeOH) extracts of *C. limon* [4, 5], aerial parts of *I. japonicus* [6], and leaves of *L. domesticum* [7, 8] showed antimutagenic effects against Trp-P-1 and PhIP. Therefore, we directed our efforts toward the isolation of their constituents and evaluation of the antimutagenic effects of the isolated constituents using the Ames test and in vivo micronucleus test*.*

## Review

### Compounds obtained from the peels of *C. limon*

The fruits of *Citrus limon* (L.) Burm.f. contain important natural chemical components, such as flavonoids, furanocoumarins, and limonoids [9]. Previous studies have described the biological activities of these compounds, such as the antioxidative, anti-inflammatory, antiallergic, antiviral, antiproliferative, anticarcinogenic, and antimutagenic activities of flavonoids [10], the suppressive effect of limonin on intestinal polyp development in *Apc*-mutant Min mice [11], and the inhibitory effects of furanocoumarins on human CYP 3A4 [12]. In addition, the essential oil of *C. limon* leaf has been shown to act as a central nervous system depressant and anticonvulsant in animal models [11].

**Fig. 1:**
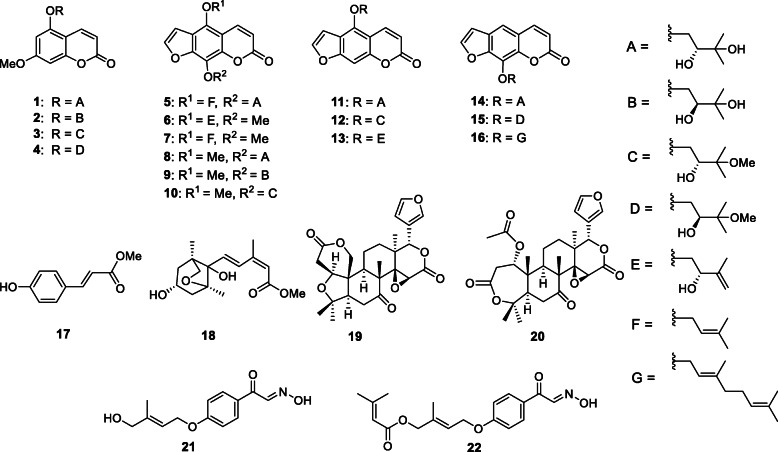
Chemical structures of the constituents isolated from *C. limon* [4, 5]

**Scheme 1:**
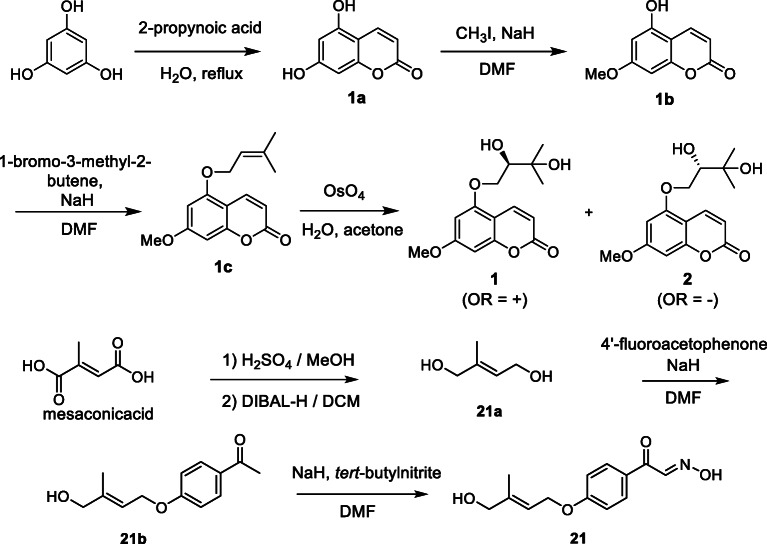
Synthesis of wakayamalimonols A (**1**), B (**2**) and limonoxime I (**21**) [4, 5].

To investigate the chemical structures of the compounds obtained from the peels of *C. limon* and their antimutagenic effects, we started the isolation using the MeOH extract obtained by refluxing the fresh peels*.* The MeOH extract was partitioned into EtOAc (ethyl acetate)- and H_2_O- soluble fractions and their antimutagenic effects were investigated using the Ames test. The EtOAc- fraction showed the potent antimutagenic effects against Trp-P-1 and PhIP. On the other hand, the H_2_O-soluble fraction showed no detectable effect. Therefore, the EtOAc-soluble fraction was subjected to normal- and reversed-phase column chromatographies, and finally HPLC to isolate four new coumarins, namely, wakayamalimonols A (**1**), B (**2**), C (**3**), and D (**4**), a new furanocoumarin wakayamalimonol E (**5**), two new oxime derivatives limonoximes I (**21**) and II (**22**), and 11 known furanocoumarins, (+)-apaensin (**6**) [13], cnidilin (**7**) [14], (+)-byakangelicin (**8**) [15], (−)-byakangelicin (**9**) [15], (+)-*tert*-*O*-methylbyakangelicin (**10**) [16], (+)-oxypeucedanin hydrate (**11**) [17], (+)-*tert*-*O*-methyloxypeucedanin hydrate (**12**) [18], (+)-pangelin (**13**) [17], (+)-2*a*,3*a*-dihydroxyimperatorin (**14**) [19], (+)-*O*-methylheraclenol (**15**) [18], and 8-geranyloxypsoralen (**16**) [20], a known phenylpropanoid, *p*-coumaric acid methylester (**17**), a known sesquiterpene, 4′-dihydrophaseric acid (**18**) [21], and two limonoids, limonin (**19**) [22], and nomilin (**20**) [23] (Fig. 1). The chemical structures of the isolated compounds were elucidated by chemical and physiochemical evidence, including NMR and MS spectra. The absolute stereochemistries of wakayamalimonol A (**1**) and B (**2**) were determined by the modified Mosher’s method. In addition, for the identification of the chemical structure and bioassay, wakayamalimonols A (**1**), B (**2**) and limonoxime I (**21**) were synthesized (Scheme 1) [4, 5].

### Compounds obtained from the aerial parts of *I. japonicus*

*Isodon japonicus* (Burm. f.) *H. Hara* (Lamiacaeae) is a perennial plant with a widely distributed in China and Japan [24]. The aerial parts of *I. japonicus* have been used as a traditional herbal medicine for the treatment of gastrointestinal disorders, tumors, and inflammatory diseases [25]. Previous reports have described the structures of diterpenoids [26], flavonoids [27], and lignans [28] as constituents of *I. japonicus*. Among them, *ent*-kaurane diterpenoids were the most characteristic constituents of *I. japonicus*. Previously, more than forty *ent*-kaurane diterpenoids were identified in phytochemical studies, and some of these compounds have cytotoxic and antibacterial activities [29]. To evaluate the antimutagenic effects of *ent*-kaurane diterpenoids, we first isolated these compounds and elucidated their structures.

**Fig. 2:**
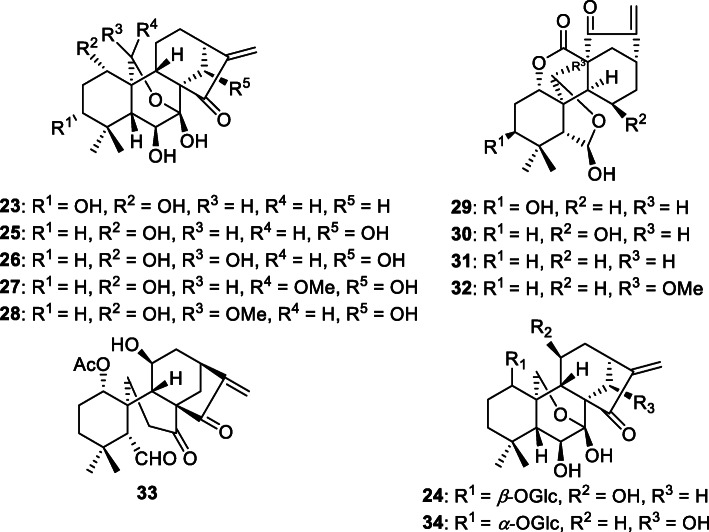
Chemical structures of the constituents isolated from *I. japonicus* [6]

From the MeOH extract of the dried aerial parts of *I. japonicus* (cultivated in Tokushima, Japan), two new *ent*-kaurane diterpenoids, named isodonterpenes I (**23**) and II (**24**), together with ten known *ent*-kaurane diterpenoids, oridonin (**25**) [30], hebeirubescensin H (**26**) [31], rabdternin F (**27**) [32], rabdternin E (**28**) [32], enmein (**29**) [33], nodosin (**30**) [34], isodocarpin (**31**) [35], serrin C (**32**) [36], isodonal (**33**) [26], and *ent*-7*β*,20-epoxy-kaur-16-ene-1*β*,6*α*,7*α*,14*α*,15*α*-pentanol-1-*O*-*β*-D-glucopyranoside (**34**) [30], were isolated (Fig. 2). The absolute stereochemistry of isodonterpene I (**23**) was elucidated by Cu-Kα X-ray crystallographic analysis [6].

### Compounds obtained from the leaves of *L. domesticum*

*Lansium domesticum* Corr. (Meliaceae), a fruit-bearing tree, grows widely in southeastern Asia [37]. The fruits of this species are edible and are very popular in desserts. Several onoceranoid-type triterpenoids have been isolated from *L. domesticum* peels [38, 39]. Previous reports have described the bioactivities of these onoceranoid-type triterpenoids, such as toxicity against brine shrimp [37], inhibition of leukotriene D4-induced contraction of the guinea pig ileum [40], cytotoxic activity [41], and antibacterial activity against Gram-positive bacteria [42]. However, the bioactivities of these onoceranoid-type triterpenoids have not been investigated thoroughly. Therefore, we attempted to isolate this class of triterpenoids for investigating their biological effects.

**Fig. 3:**
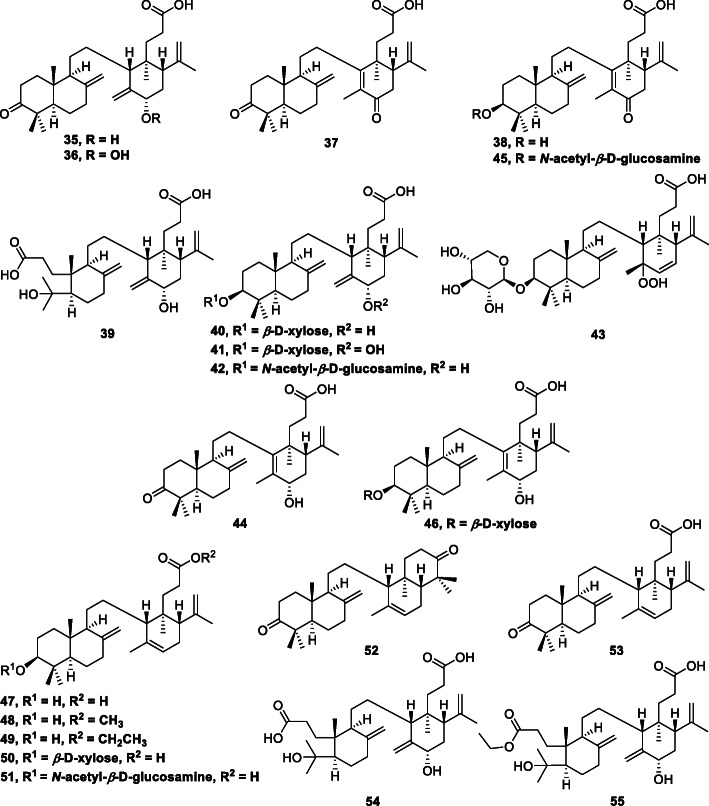
Chemical structures of the constituents isolated from *L. domesticum* [7, 8]

From the MeOH extract of *Lansium domesticum* dried leaves, twelve new compounds, namely, lansium acids I (**35**), II (**36**), III (**37**), IV (**38**), V (**39**), VI (**40**), VII (**41**), VIII (**42**), IX (**43**), X (**44**), XI (**45**), and XII (**46**) together with nine known compounds, lansiolic acid (**47**) [40], methyl lansiolate (**48**) [40], ethyl lansiolate (**49**) [42], lansioside C (**50**) [40], lansioside B (**51**) [40], 8,14-*seco*-gammacera-7,14-diene-3,21-dione (**52**) [43], lansionic acid (**53**) [37], lansic acid (**54**) [37], and lamesticumin A (**55**) [42] were isolated (Fig. 3). The absolute stereo structures of the new compounds were established by comparison of the experimental and predicted electronic circular dichroism data [7, 8].

### Evaluation of the antimutagenic effects of isolated compounds using the Ames test

The antimutagenic effects of the isolated compounds were evaluated against Trp-P-1 and PhIP by the Ames test using the *S. typhimurium* TA98 strain (Tables 1, 2, 3 and 4). Trp-P-1 and PhIP are well known mutagenic and carcinogenic heterocyclic amines found in cooked meat. We used nobiletin as the positive control that have been reported to have antimutagenic effects using the Ames test [44]. As shown in Tables 2 and 4, among the compounds isolated from the peels of *C. limon*, furanocoumarins, (+)-*tert*-*O*-methyloxypeucedanin hydrate (**12**) [inhibition against Trp-P-1: 74.7% at 50 nmol/plate] and (+)-2*a*,3*a*-dihydroxyimperatorin (**14**) [inhibition against Trp-P-1: 73.7% at 50 nmol/plate] showed potent antimutagenic effects. The antimutagenic effects of **12** and **14** were much stronger than that of the reference compound, nobiletin [inhibition against Trp-P-1: 56.0% at 80 nmol/plate]. On the other hand, coumarins (**1** and **2**), and limonoids (**19** and **20**) showed weak but dose-dependent antimutagenic effects against Trp-P-1 and PhIP. The oxime (**21**) and synthetic intermediate (**21b**) showed antimutagenic effect, but the synthetic intermediate without phenyl group (**21a**) did not show any effects. These results suggest that a phenyl group is necessary for the antimutagenic effects of this class of compounds [4, 5]. The *ent*-kaurane diterpenoids (**26**, **27**, **30, 31**, and **34**) from *I. japonicus* also showed antimutagenic effects against Trp-P-1 [inhibition against Trp-P-1: 47.4–57.3% at 100 nmol/plate]. While their effects were not stronger than that of nobiletin, our study represented the first evaluation of the antimutagenic activities of *ent*-kaurane diterpenes [6]. Among the isolated onoceranoid-type triterpenoids, the antimutagenic effects of lansiolic acid (**47**) [inhibition against Trp-P-1: 73.8% at 100 nmol/plate] and lansionic acid (**53**) [inhibition against Trp-P-1: 79.9% at 100 nmol/plate] against Trp-P-1 were equivalent to that of nobiletin. In addition, interesting structure-activity relationships were suggested. Specifically, the compound with a carboxylic acid moiety (**47**) showed more potent antimutagenic effects than its analogs with either a methyl ester moiety (**48**) [inhibition against Trp-P-1: 29.5% at 100 nmol/plate] or ethyl ester moiety (**49**) [inhibition against Trp-P-1: 7.9% at 100 nmol/plate]. Among the esters, the ethyl ester compound **49** showed weaker effects than the methyl ester **48** [7, 8]. The antibacterial activity of tested compounds against the *S. typhimurium* TA98 strain were tested using nutrient agar containing NaCl and all compounds showed weak [**44:** 23.2% inhibition at 400 nmol/plate, **54**: 23.0% inhibition at 400 nmol/plate] or no (other compounds: < 5.0% inhibition at highest concentration in Table 1) antibacterial activities [4–8].

**Table 1:** Antimutagenic activity of coumarins (**1** and **2**), limonoids (**19** and **20**), oxime and synthetic intermediates (**21**, **21a**, and **21b**), *ent*-kaurane diterpenoids (**25**, **26**, **29**, **30**, and **33**), and onoceranoid-type triterpenoids (**44**, **46**–**49**, **53**, and **54**) against Trp-P-1 (0.04 μg/plate) using the Ames test [4–8]^*a*^

	Inhibition % (Based on number of revertant colonies)				
	50 nmol/plate	100 nmol/plate	200 nmol/plate	400 nmol/plate	800 nmol/plate
Coumarins					
**1**^*b*^	45.8%	58.7%	64.6%	75.6%	82.3%
**2**^*b*^	57.8%	59.8%	67.1%	77.2%	81.4%
Limonoids					
**19**^*b*^	10.7%	8.0%	31.0%	46.0%	45.8%
**20**^*b*^	38.7%	54.9%	69.8%	83.1%	
Oxime					
**21**^*b*^	27.3%	26.2%	40.5%	53.7%	49.0%
Synthetic intermediates of **21**					
**21a**^*b*^	22.2%	20.6%	21.6%	0.2%	–
**21b**^*b*^	31.7%	39.2%	51.7%	54.7%	61.4%
*ent*-Kaurane diterpenoids					
**25**^*b*^	34.6%	57.3%	53.7%	65.2%	
**26**^*b*^	32.9%	54.4%	60.9%	67.8%	
**29**^*b*^	21.0%	47.5%	52.0%	59.9%	
**30**^*b*^	46.0%	56.8%	72.8%	73.3%	
**33**^*b*^	41.0%	54.9%	68.3%		
Onoceranoid-type triterpenoids					
**44**^*c*^	11%	25%	50%	88%	
**46**^*b*^	14%	23%	41%	65%	
**47**^*b*^	–	73.8%	90.5%	94.2%	
**48**^*b*^	0.4%	29.5%	64.8%	77.5%	
**49**^*b*^	3.7%	7.9%	8.7%	42.6%	
**53**^*b*^	5.1%	79.9%	91.5%	92.4%	
**54**^*c*^	–	25.0%	61.0%	93.0%	

^*a*^ Nobiletin was used as a reference compound. It showed 56% inhibition at 20 nmol/plate

^*b*^ Compounds showed no antibacterial activity [< 5.0% inhibition at highest concentration] against the *S. typhimurium* TA98 strain

^*c*^ Compounds showed weak antibacterial activity [**44:** 23.2% inhibition at 400 nmol/plate, **54**: 23.0% inhibition at 400 nmol/plate] against the *S. typhimurium* TA98 strain

**Table 2:** Antimutagenic activity of furanocoumarins (**11**, **12**, **14**, and **15**) against Trp-P-1 (0.04 μg/plate) using the Ames test [4]^*a*^

	Inhibition % (Based on number of revertant colonies)				
	3.1 nmol/plate	6.3 nmol/plate	12.5 nmol/plate	25 nmol/plate	50 nmol/plate
Furanocoumarins					
**11**	22%	13.9%	25.0%	31.5%	40.9%
**12**	27.0%	35.9%	51.6%	59.5%	74.7%
**14**	25.0%	40.5%	49.7%	62.8%	73.7%
**15**	5.6%	5.6%	18.1%	36.6%	51.2%

^*a*^ No compound showed antibacterial activity [< 5.0% inhibition at highest concentration] against the *S. typhimurium* TA98 strain at the tested concentrations

**Table 3:** Antimutagenic activity of coumarins (**1** and **2**), limonoids (**19** and **20**), oxime and synthetic intermediates (**21**, **21a**, and **21b**), *ent*-kaurane diterpenoids (**25**, **26**, **29**, **30**, and **33**), and onoceranoid-type triterpenoids (**47**–**49**, **53**, and **54**) against PhIP (1.0 μg/plate) using the Ames test [4–8] ^*a,b*^

	Inhibition % (Based on number of revertant colonies)				
	50 nmol/plate	100 nmol/plate	200 nmol/plate	400 nmol/plate	800 nmol/plate
Coumarins					
**1**^*b*^	17.5%	29.1%	48.4%	48.6%	81.4%
**2**^*b*^	18.2%	35.4%	62.2%	77.6%	86.3%
Limonoids					
**19**^*b*^	27.0%	39.0%	25.5%	50.3%	46.7%
**20**^*b*^	23.6%	44.2%	74.9%	79.8%	
Oxime					
**21**^*b*^	12.2%	11.1%	29.7%	47.1%	63.7%
Synthetic intermediates of **21**					
**21a**^*b*^	8.6%	7.4%	0.7%	–	–
**21b**^*b*^	10.7%	18.0%	39.0%	53.3%	68.1%
*ent*-Kaurane diterpenoids					
**25**^*b*^	55.8%	62.8%	73.7%	80.7%	
**26**^*b*^	54.3%	72.0%	79.5%	85.5%	
**29**^*b*^	24.0%	43.2%	62.3%	74.2%	
**30**^*b*^	51.0%	64.8%	81.4%	85.5%	
**33**^*b*^	71.0%	79.5%	80.4%		
Onoceranoid-type triterpenoids					
**47**^*b*^	30.8%	72.7%	93.7%	94.9%	
**48**^*b*^	14.3%	33.7%	54.3%	74.4%	
**49**^*b*^	4.1%	17.2%	22.5%	41.0%	
**53**^*b*^	36.8%	65.2%	94.1%	93.9%	
**54**^*c*^	18.6%	32.4%	63.2%	95.4%	

^*a*^ Nobiletin was used as a reference compound. It showed 56% inhibition at 20 nmol/plate

^*b*^ Compounds showed no antibacterial activity [< 5.0% inhibition at highest concentration] against the *S. typhimurium* TA98 strain

^*c*^ Compound showed weak antibacterial activity [**54**: 23.0% inhibition at 400 nmol/plate] against the *S. typhimurium* TA98 strain

**Table 4:** Antimutagenic activity of furanocoumarins (**11**, **12**, **14**, and **15**) against PhIP (1.0 μg/plate) using the Ames test [4]^*a*^

	Inhibition % (Based on number of revertant colonies)				
	3.1 nmol/plate	6.3 nmol/plate	12.5 nmol/plate	25 nmol/plate	50 nmol/plate
Furanocoumarins					
**11**	22.0%	28.0%	29.7%	46.3%	64.0%
**12**	47.0%	57.7%	68.5%	73.4%	83.4%
**14**	34.0%	41.7%	51.7%	67.6%	70.1%
**15**	47.1%	62.1%	65.3%	69.6%	78.7%

^*a*^ No compound showed antibacterial activity [< 5.0% inhibition at highest concentration] against the *S. typhimurium* TA98 strain at the tested concentrations

### Evaluation of antimutagenic effects of limonin (19) and lansionic acid (53) using in vivo micronucleus test

**Fig. 4:**
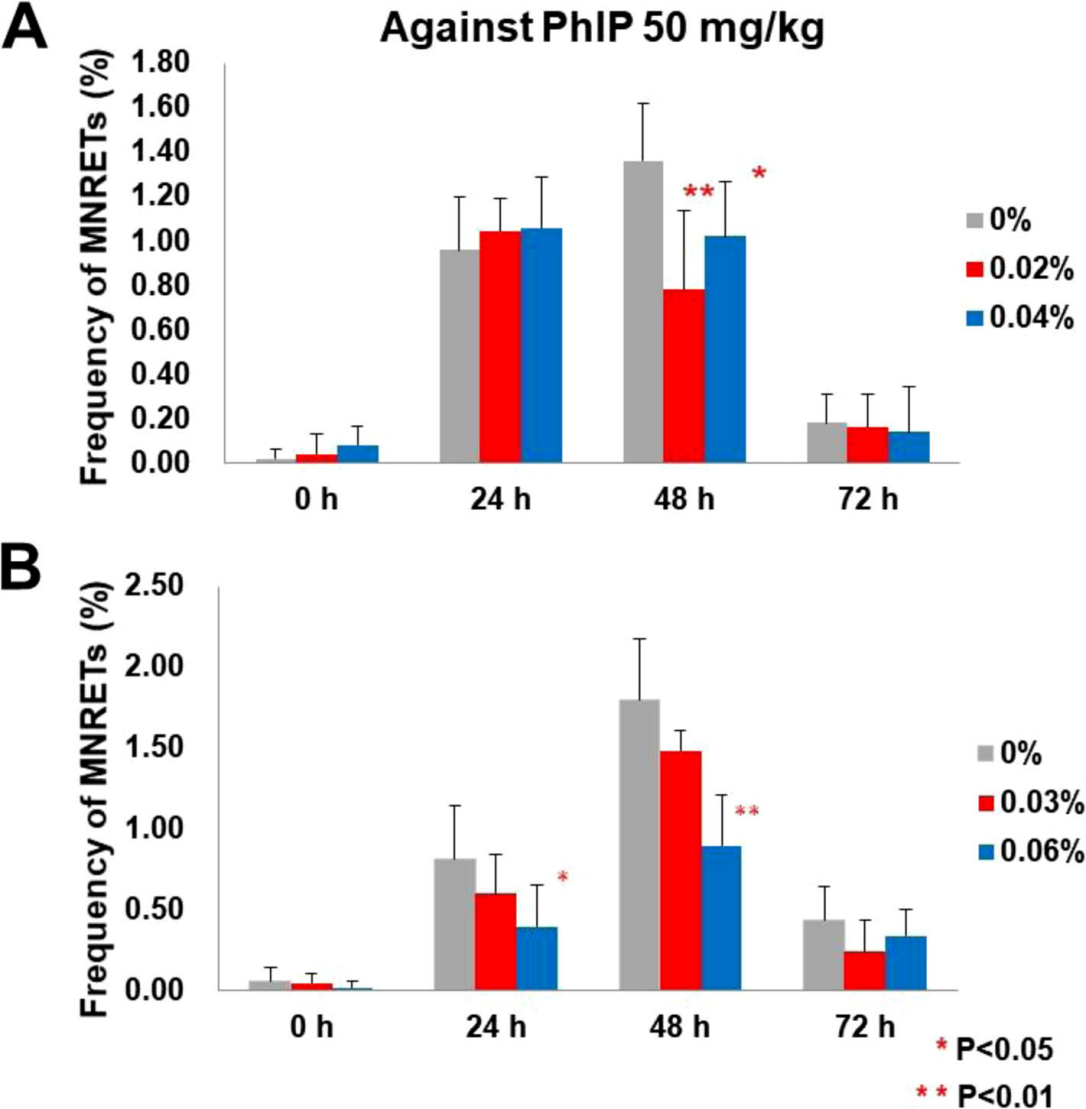
Frequency of MNRETs from peripheral blood of mice treated with mutagen {PhIP (50 mg/kg bw)}. Significant difference: *0.01 < *P* < 0.05; **P < 0.01 (Student’s *t* test). Each point represents the mean and standard deviation of five mice. **a** Normal or sample feeds that included limonin (**19**) at low or high dose (0.02% or 0.04%, w/w) were given ad libitum. **b** Normal or sample feeds that included lansionic acid (**53**) at low or high dose (0.03% or 0.06%, w/w) were given ad libitum [4, 8]

To examine the antimutagenic effects of limonin (**19**) and lansionic acid (**53)** in vivo, we conducted a micronucleus test using the peripheral blood of male ICR mice. These two compounds were isolated enough amount and are the major constituents of *C. limon* and *L. domesticum.* The micronucleus test detects chromosomal damage induced by genotoxic/carcinogenic compounds, and it has been used to evaluate antimutagenic agents in vivo. We gave either normal feed or sample feed that included the limonin (**19**) or lansionic acid (**53)** at low or high dose. The mouse tail vein blood (5 μL) was taken prior to the administration of PhIP, and at 24, 48, and 72 h after administration. As a result, limonin (**19**) and lansionic acid (**53)** significantly decreased the frequency of micronucleated reticulocytes (MNRETs) treated with PhIP at 24 h and 48 h after the administration (Fig. 4) [4, 7].

## Conclusions

In conclusion, twenty-one new compounds and thirty-four known compounds including coumarins, furanocoumarins, oximes, *ent*-kaurane diterpenoids, and onoceranoid-type triterpenoids were isolated from the peels of *C. limon*, aerial parts of *I. japonicus*, and leaves of *L. domesticum*. Among them, furanocoumarins showed the strongest antimutagenic effects in the Ames test. It is important to note that furanocoumarins were well known Cytochrome P450 (CYP) 1A2 inhibitors [45], and the mutagenicity of Trp-P-1 and PhIP depends on their bioactivation by CYP 1A2 [46]. These facts suggest that one of the mechanisms of the antimutagenic effects of furanocoumarins against Trp-P-1 and PhIP may be the inhibition of the CYP 1A2 enzyme. In addition, the oral intake of limonin (**19**) and lansionic acid (**53**), which are the major constituents of *C. limon* and *L. domesticum*, respectively, showed significant antimutagenic effects against PhIP in the in vivo micronucleus test. These results suggest that these compounds and plant materials are likely useful agents for cancer prevention.

## Data Availability

Not applicable.

## Abbreviations

ECD: Electronic circular dichroism
HPLC: high-performance liquid chromatography
ICR: Institute of Cancer Research
MNRET: micronucleated reticulocyte
NMR: nuclear magnetic resonance
Trp-P-1: 3-amino-1,4-dimethyl-5*H*-pyrido[4,3-*b*]indole
PhIP: 2-amino-1-methyl-6-phenylimidazo[4,5-*b*]pyridine

## References

[1] Nagao M, Tsugane S (2016). Cancer in Japan: prevalence, prevention and the role of heterocyclic amines in human carcinogenesis. Genes Environ.

[2] Izquierdo-Vega JA, Morales-González JA, SánchezGutiérrez M, Betanzos-Cabrera G, Sosa-Delgado SM, Sumaya-Martínez MT, Morales-González Á, Paniagua-Pérez R, Madrigal-Bujaidar E, Madrigal-Santillán E (2017). Evidence of some natural products with Antigenotoxic effects. Part 1: fruits and polysaccharides. Nutrients.

[3] Li WQ, Kuriyama S, Li Q, Nagai M, Hozawa A, Nishino Y, Tsuji I (2010). Citrus consumption and cancer incidence: the Ohsaki cohort study. Int J Cancer.

[4] Matsumoto T, Takahashi K, Kanayama S, Nakano Y, Imai H, Kibi M, Imahori D, Hasei T, Watanabe T (2017). Structures of antimutagenic constituents in the peels of *Citrus limon*. J Nat Med.

[5] Matsumoto T, Koike M, Arai C, Kitagawa T, Inoue E, Imahori D, Watanabe T (2018). Chemical structures and antimutagenic effects of unusual oximes from the peels of *Citrus limon*. Phytochem Lett.

[6] Matsumoto T, Nakamura S, Kojima N, Hasei T, Yamashita M, Watanabe T, Matsuda H (2017). Antimutagenic activity of *ent*-kaurane diterpenoids from the aerial parts of *Isodon japonicus*. Tetrahedron Lett.

[7] Matsumoto T, Kitagawa T, Teo S, Anai Y, Ikeda R, Imahori D, Ahmad HS, Watanabe T (2018). Structures and antimutagenic effects of onoceranoid-type triterpenoids from the leaves of *Lansium domesticum*. J Nat Prod.

[8] Matsumoto T, Kitagawa T, Ohta T, Yoshida T, Imahori D, Stephen T, Ahmad HS, Watanabe T (2019). Structures triterpenoids from the leaves of *Lansium domesticum*. J Nat Med.

[9] Bruno L, Spadafora ND, Iaria D, Chiappetta A, Bitonti MB (2014). Developmental stimuli and stress factors affect expression of ClGLP1, an emerging allergen-related gene in *Citrus limon*. Plant Physiol Biochem.

[10] Campelo LM, Goncalves FC, Feitosa CM, Freitas RM (2011). Antioxidant activity of *Citrus limon* essential oil in mouse hippocampus. Pharm Biol.

[11] Shimizu S, Miyamoto S, Fujii G, Onuma W, Ozaki Y, Fujimoto K, Yano T, Mutoh M (2015). Suppression of intestinal carcinogenesis in Apc-mutant mice by limonin. J Clin Nutr.

[12] Girennavar B, Poulose SM, Jayaprakasha GK, Bhat NG, Patil BS (2006). Furocoumarins from grapefruit juice and their effect on human CYP 3A4 and CYP 1B1 isoenzymes. Bioorg Med Chem.

[13] Liu DP, Luo Q, Wang GH, Xu Y, Zhang XK, Chen QC, Chen HF (2011). Furocoumarin derivatives from radix angelicae dahuricae and their effects on RXRα transcriptional regulation. Molecules.

[14] Franke K, Porzel A, Masaoud M, Adam G, Schmidt J (2001). Furanocoumarins from *Dorstenia gigas*. Phytochemistry.

[15] Fujioka T, Furumi K, Fujii H, Okabe H, Mihashi K, Nakano Y, Matsunaga H, Katano M, Mori M (1999). Antiproliferative constituents from umbelliferae plants. V A new furanocoumarin and falcarindiol furanocoumarin ethers from the root of Angelica japonica. Chem Pharm Bull.

[16] Mendez J, Poceiro JC (1982). Furocoumarins from *Angelica pachycarpa*. Phytochemistry.

[17] Thanh PN, Jin WY, Dong GY, Bae K, Kang SS (2004). Cytotoxic coumarins from the root of *Angelica dahurica*. Arch Pharm Res.

[18] Seo WD, Kim JY, Ryu HW, Kim JH, Han SI, Ra JE, Seo KH, Jang KC, Lee JH (2013). Identification and characterisation of coumarins from the roots of *Angelica dahurica* and their inhibitory effects against cholinesterase. J Funct Foods.

[19] Lv X, Liu D, Hou J, Dong P, Zhan L, Wang L, Deng S, Wang C, Yao J, Shu X, Liu L, Ma X (2013). Biotransformation of imperatorin by *Penicillium janthinellum*. Anti-osteoporosis activities of its metabolites. Food Chem.

[20] Row EC, Brown SA, Stachulski AV, Lennard MS (2006). Synthesis of 8-geranyloxypsoralen analogues and their evaluation as inhibitors of CYP3A4. Bioorg Med Chem.

[21] Takasugi M, Anetai M, Katsui N, Masamune T (1973). The occurrence of vomifoliol, dehydrovomifoliol, and dihydrophaseic acid in the roots of “kidney bean”. Chem Lett.

[22] Sugimoto T, Ueno A, Kadota S, Cui C, Kikuchi T (1988). New 5β-H limonoids from *Evodia rutaecarpa* Bentham. Chem Pharm Bull.

[23] Liu J (2001). Two new limonoids from *Polygonum orientaleI* L. Indian J Chem.

[24] Lim YJ, Won JT, Hwang YB, Kim RH, Hwang WK, Sul D, Park YS (2010). The new diterpene isodojaponin D inhibited LPS-induced microglial activation through NF-kappaB and MAPK signaling pathways. Eur J Pharmacol.

[25] Tanaka T, Nakashima T, Ueda T, Tomii K, Kouno I (2007). Facile discrimination of aldose enantiomers by reversed-phase HPLC. Chem Pharm Bull.

[26] Takeda Y, Fujita T, Sun DH, Minami Y, Ochi M, Cheen CC (1990). Revision of the structures of isodonal, rabdolasional and related diterpenoids. Chem Pharm Bull.

[27] Hong SS, Lee C, Lee HC, Park M, Lee SM, Hong TJ, Lee H, Lee KM, Hwang YB (2009). A new furofuran lignan from *Isodon japonicus*. Arch Pharm Res.

[28] Agata I, Hatano T, Nishibne S, Okuda T (1989). A tetrameric derivative of caffeic acid from Rabdosia japonica. Phytochemistry.

[29] Hou JR, Yan LF, Zhang YH, Bai XY, Ding MM (2012). Two new diterpenoids and other constituents from Isodon japonicus. Helv Chim Acta.

[30] Liu HM, Yan X, Kikuchi F, Liu Z (2000). A new diterpene glycoside from *Rabdosia rubescens*. Chem Pharm Bull.

[31] Huang SX, Zhou Y, Pu JX, Li RT, Xiao WL, Lou LG, HanQB DLS, Peng SL, Sun HD (2006). Cytotoxic *ent*-kauranoid derivatives from *Isodon rubescens*. Tetrahedron.

[32] Takeda Y, Ikeda K, Fujita T, Han-dong S, Minami Y (1994). Rabdoternins D-G, *ent*-7*β*,20-epoxykaurenes from *Rabdosia ternifolia*. Phytochemistry.

[33] Li LM, Li GY, Pu JX, Xiao WL, Ding LS, Sun HD (2009). *ent*-Kaurane and cembrane diterpenoids from *Isodon sculponeatus* and their cytotoxicity. J Nat Prod.

[34] Fujita E, Fujita T, Shibuya M (1968). Terpenoids. VII. The structure and absolute configuration of nodosin, a new diterpenoid from Isodon species. Chem Pharm Bull.

[35] Fujita E, Fujita T, Shibuya M (1968). Terpenoids. IX. The structure and absolute configuration of Isodocarpin, a new diterpenoid from *Isodon trichocarpus* KUDO and *I. japonicus* HARA. Chem Pharm Bull.

[36] Zhao AH, Zhang Y, Xu ZH, Liu JW, Jia W (2004). Immunosuppressive *ent*-Kaurene diterpenoids from *Isodon serra*. Helv Chim Acta.

[37] Tanaka T, Ishibashi M, Fujimoto H, Okuyama E, Koyano T, Kowithayakorn T, Hayashi M, Komiyama K (2002). New onoceranoid triterpene constituents from *Lansium domesticum*. J Nat Prod.

[38] Mayanti T, Tjokronegoro R, Supratman U, Mukhtar MR, Awang K, Hadi AH (2011). Antifeedant triterpenoids from the seeds and bark of *Lansium domesticum* cv Kokossan (Meliaceae). Molecules.

[39] Saewan N, Sutherland JD, Chantrapromma K (2006). Antimalarial tetranortriterpenoids from the seeds of *Lansium domesticum* Corr. Phytochemistry.

[40] Nishizawa M, Nishida H, Kosela S, Hayashi Y (1983). Structure of lansiosides: biologically active new triterpene glycides from *Lansium domesticum*. J Organomet Chem.

[41] Nugroho AE, Inoue D, Wong CP, Hirasawa Y, Kaneda T, Shirota O, Hadi AHA, Morita H (2018). Reinereins a and B, new onocerane triterpenoids from Reinwardtiodendron cinereum. J Nat Med.

[42] Dong SH, Zhang CR, Dong L, Wu Y, Yue JM (2011). Onoceranoid-type triterpenenoids from *Lansium domesticum*. J Nat Prod.

[43] Habaguchi K, Watanabe M, Nakadaira Y, Nakanishi K (1968). The full structures of lansic acid and its minor congener, an unsymmetric onoceradienedione. Tetrahedron Lett.

[44] Okuno Y, Miyazawa M (2004). Biotransformation of nobiletin by Aspergillus Niger and the antimutagenic activity of a metabolite, 4′-hydroxy-5,6,7,8,3′-pentamethoxyflavone. J Nat Prod.

[45] Olguin-Reyes S, Camacho-Carranza R, Hernandez-Ojeda S, Elinos-Baez M, Espinosa-Aguirre JJ (2012). Bergamottin is a competitive inhibitor of CYP1A1 and is antimutagenic in the Ames test. Food Chem Toxicol.

[46] Chevereau M, Glatti H, Zalko D, Cravedi JP, Audebert M (2017). Role of human sulfotransferase 1A1 and N-acetyltransferase 2 in the metabolic activation of 16 heterocyclic amines and related heterocyclics to genotoxicants in recombinant V79 cells. Arch Toxicol.

